# Development and evaluation of clove oil nanoemulsion-based topical cream for anti-inflammatory activity in mice

**DOI:** 10.3389/fvets.2025.1716637

**Published:** 2025-12-17

**Authors:** Ahsan Shafiq, Irfan Baboo, Zahid Farooq, Hamid Majeed, Valiollah Palangi

**Affiliations:** 1 Cholistan Institute of Biological Sciences, Cholistan University of Veterinary and Animal Sciences (CUVAS), Bahawalpur, Pakistan; 2 Department of Food Science and Technology, Cholistan University of Veterinary and Animal Sciences (CUVAS), Bahawalpur, Pakistan; 3 Department of Animal Science, Faculty of Agriculture, Near East University, Nicosia, Northern Cyprus, Türkiye

**Keywords:** anti-inflammatory, nanoemulsion, topical cream, drug release, sustainability

## Abstract

**Introduction:**

Inflammatory skin disorders require effective topical therapies with minimal side effects. Clove (Syzygium aromaticum) is recognized for its potent anti-inflammatory, antimicrobial, and antioxidant properties, but it has a limited clinical use due to its highly volatile nature, poor solubility, and potential skin irritation at higher concentrations. This research aimed to develop and optimize clove oil nanoemulsion (CONE)-based topical cream, characterize its physicochemical properties, and evaluate anti-inflammatory efficacy using a mouse model.

**Methodology & Results:**

CONE was prepared via ultrasonication and optimized using response surface methodology. The optimized CONE exhibited a mean droplet size of approximately 190 nm and Polydispersity Index of 0.08, with high entrapment efficiency (94.54%). GC-MS analysis confirmed eugenol as the major constituent. The nanoemulsion demonstrated strong antifungal activity. The minimum inhibitory concentration was 120 μl/mL. CONE significantly enhanced antioxidant capacity compared to clove oil. The cream was formulated by incorporating CONE into carboxymethyl cellulose (CMC) matrix and evaluated for stability, pH, morphology, and drug release. The cream maintains stability, favorable organoleptic properties, and sustained drug release, particularly at a 1 mL CONE concentration. Thirty adult male albino mice (30–40g) were used and randomly divided into six groups. Hematological parameters and C-reactive protein level further supported the anti-inflammatory efficacy topical cream, with marked improvements observed in treated groups. Histopathological analysis revealed re-epithelialization and diminished inflammatory infiltration.

**Conclusion:**

CONE-based cream offers a promising, safe, and effective topical therapy for inflammatory skin conditions. The nanoemulsion formulation enhances clove oil’s bioavailability, stability, and therapeutic potential, supporting further development for clinical and cosmetic applications.

## 1 Introduction

Inflammatory skin disorders are a major health concern, often requiring long-term management with topical agents (1). Inflammation is the biological process the body develops in response to tissue damage or infection (2). It is a body’s vascular and cellular reaction to an irritating incursion. It is well-defined by a combination of cardinal signs: swelling, redness, pain, and the symptom of infection or injury (3). In the absence of inflammation, infections, wounds, and tissue damage are not resolved (4). Conventional treatment modes of treating inflammatory dermatoses majorly rely on the use of corticosteroids and non-steroidal anti-inflammatory effects (5). All drugs used in treating various inflammatory diseases have long-term negative effects (6, 7). These limitations have prompted a growing interest in natural bioactive compounds.

As plant secondary metabolites, essential oils (EO) have recently emerged as therapeutics for inflammation and pain (8). The natural essentials oil are safe and effective substitutes to the standard curative treatments, such as clove oil. Clove is an aromatic flowering bud that is cultivated in various parts of the world for medical, culinary, and perfumery purposes (1, 9). *Syzygium aromaticum* contains approximately 15–20% of EO. Clove essential oil (CEO) is rich in phenolic compounds that have several biological properties, such as antibacterial, anti-inflammatory, antioxidant, analgesic, and insecticidal properties (10, 62). Clove oil supports wound healing by enhancing fibroblastic migration, wound healing, increasing angiogenesis, as well as alleviating scar formation (11). The bioactives can degrade when subjected to light, oxygen, variation in temperature, and moisture (12). As a solution to such instability, clove oil can be incorporated into nanoscale carrier systems hence maintaining its integrity and maximizing its delivery. Despite its therapeutic potential, the clinical application of clove oil in topical formulations is hampered by its volatility, poor aqueous solubility, and potential for skin irritation at higher concentrations (13).

Preparation of EOs as nanoformulations is a hopeful method to improve their action (14). Amongst the nanoformulations, nanoemulsions are highly considered because of the lower side effects and bioavailability, especially easier preparation (15, 16). NEs are biphasic clear dispersions that consist of an oil and water phase that are stably combined using surfactants/co-surfactants. Due to their droplet size, below 200 nm, such systems remain stable droplets against aggregation or creaming (17).

Effective topical delivery of nanoemulsions has some promising features, such as increased skin permeability and no irritation (18). The conversion of nanoemulsions to gels enhances the topical applications, physical stability, and heat resistance for EOs (19). Topical drug delivery is a critical factor in the therapeutic treatment of various cutaneous pathologies that include infectious dermatoses, cutaneous neoplasma, dermatitis, burn, inflammation, chronic wounds, as well as psoriasis (20). Nanoemulsion-based topical creams represent a more sophisticated approach to drug delivery. The diminutive droplet size significantly improves the solubility and bioavailability of poorly water-soluble drugs (21). Nanoemulsions enhance skin permeability and provide controlled release of the active ingredient constitutes a major advantage over conventional formulations (22). Consequently, nanoemulsion-based creams are increasingly being investigated for the delivery of a wide range of therapeutic agents, including antimicrobial, anti-inflammatory and antioxidant compounds (23). The aim of the conducted study was to design a topical cream based on nanoemulsions, to optimize and evaluate the characteristics of the topical cream that contains clove oil and has the aim of testing its anti-inflammatory properties in a mice model. The nanoemulsion will provide a novel, effective, and safe topical anti-inflammatory drug delivery system, which might make the best use of clove oil.

## 2 Materials and methods

Clove oil and canola oil were purchased from Karachi Essence, Lahore (Pakistan). Potato Dextrose Agar (PDA) and Tween80 were purchased from Sigma Aldrich (Saint Louis, MO, USA).

### 2.1 Preparation and optimization of nanoemulsion

Preparation and optimization of nanoemulsion was done by the ultrasonication method as reported by Jabbar et al. (24). The optimization process of CONE was performed by response surface methodology (RSM) in Box–Behnken Design (BBD) to improve the droplet size and Polydispersity Index (PDI). Tween80 aqueous solution (2.5% w/v) was prepared in deionized water at room temperature, followed by stirring for 30 min at 40 °C. Addition of clove oil (mixed phase 10%) and homogenized at 18000 rpm for 5 min using DH-1500 ultra turrax homogenizer. The crude emulsion was homogenized using an Ultra-Sonicator (UCD-1200, BIOBASE, Jinan, China) for 10 min at 50% power (30:30 s rest/work cycle). During ultrasonication, the samples were kept in a beaker containing ice water to minimize heat loss. The prepared nanoemulsion formulations were all kept at 4 °C until use for the experiments.

### 2.2 Characterization of nanoemulsion

Mean droplet size and PDI were determined by the dynamic light scattering (DLS) technique by diluting 200 times with fresh deionized water using zeta sizer Nano ZS 90 (25).

### 2.3 Gas chromatography–mass spectrometry (GC–MS) analysis

The clove oil was analyzed by GC–MS according to the method of Kelidari et al. (26), using a series of 7890A Network GC interfaced with a 5975C VL MSD with Triple-Axis Detector (Agilent Technologies, Santa Clara, CA, USA). A HP-5MS fused silica column was used for component separation, and the initial temperature was set at 40 °C for 1 min, and then the temperature was increased to the final temperature of 250 °C at a rate of 3 °C/min held for 90 min (80). Split-flow 10 mL/min with 6 mL/min septum purge, and 1 mL/min column flow. The helium gas was used as the carrier gas and it had a purity of 99.99%.

### 2.4 Transmission electron microscopy (TEM)

The morphological study of nanoemulsion droplets was thoroughly inspected through the use of digital transmission electron microscopy imaging. The CONE emulsion was deposited onto a carbon film laid upon a copper mesh utilizing a micropipette. It was then stained for contrast with a solution of 2% weight by volume phosphotungstic acid. Before analysis under the transmission electron microscope, the sample-coated mesh was permitted to thoroughly dehydrate in ambient conditions for a full day. Next, the TEM, operating at 100 KV, was employed to examine the stained specimen. This provided high-resolution images of droplet structure, allowing for a comprehensive analysis of NE morphology according to the methods outlined by Majeed et al. (27).

### 2.5 Entrapment efficiency of nanoemulsion

The entrapment efficiency of CONE was determined as described earlier by Majeed et al. (28), and Heydari et al. (29). Briefly, the nanoemulsions were mixed in distilled water under vigorous stirring for 10 min. The sample was centrifuged to get the supernatant, which contained unencapsulated essential oil. The sediment was mixed with water and later ethanol was added till further vortex for 15 min to achieve the inner essential oil content. The efficiency was calculated using the expression (Equation 1).


$$\text{Entrapment Efficiency}=[\frac{\left(\text{External}-\text{Internal}\right)}{\left(\text{Total loaded}\right)}]\times 100$$ (1)

### 2.6 Formulation of cream

Carboxymethyl cellulose polymer (CMC) was selected for its frequent use in cosmetic fields (30). CMC powder (2 g) was dissolved in 100 mL distilled water, and stirring was conducted at 60 °C by a heater-stirrer (RTH-340, Robus Technologies, UK) for 45–60 min. The dissolved solution of CMC polymer was incorporated directly with different concentrations (w/v) of nanoemulsion and stirred for 10 min. Four different concentrations of clove oil nanoemulsion were used in the formulation of cream (500 μL, 750 μL, 1 mL, and 1.5 mL). Each concentration was added to 6 g of CMC solution. The formulated creams were evaluated for physical properties and further evaluation.

### 2.7 Organoleptic properties of cream

The appearance of the cream is judged by its color, smell, and texture (31).

#### 2.7.1 pH of cream

The pH of the formulated cream was determined with a digital pH meter (32).

#### 2.7.2 Cream separation

Cream (5 g) was centrifuged at 5000 rpm for 10 min at 25 °C (33).

#### 2.7.3 Cream stability

The stability was tested in various conditions of the heating–cooling cycle (6 cycles): 45 °C (in incubator) and 4 °C (in refrigerator) for 48 h each cycle (34).

### 2.8 Entrapped drug release

An evaluation of the entrapped drug release of NE was conducted using UV spectroscopy. Ethanol was used to dissolve the best-formulated cream incorporating the CONE.

### 2.9 Antifungal efficacy

The agar-dilution method was applied to test the antifungal activity of CONE against *Aspergillus niger* and *Penicillium italicum*. The antifungal test was carried out according to the microdilution technique in 96-well plates, as reported earlier by Song et al. (35), with a few modifications. The examined levels of nanoemulsion were 20, 40, 60, 80, 100, 120, and 140 μL/mL. The fungal isolates were grown in potato dextrose agar (PDA) medium. The plates (96 wells) were incubated for 30 min at room temperature before 10ul of the fungal strains at 10^4^ CFU/mL was added. Absorbance was measured at 340 nm. The time-kill graphs of fungi against CONE at their MIC were evaluated by following Majeed et al. (36). Time kills were performed at 0, 6, 12, 24, 36, 48, and 72 h by using the microdilution method in 96-well plates.

### 2.10 Antioxidant activity

#### 2.10.1 DPPH radical capture assay

The antioxidant property of the CONE was evaluated by the DPPH method (2,2-diphenyl-1-picrylhydrazyl) (37). The CONE samples (10, 20, 40, 60, 80, 100 and 120 μL) were diluted in water to avoid aggregation. The CONE samples were added to 1 mL of methanolic DPPH solution (0.3 mmol) and incubated at 30 °C for 30 min in the dark; absorbance was measured at 517 nm on a UV–VIS spectrophotometer (T80, United Kingdom) (82). The scavenging rate was measured as the following Equation 2:


$$\text{Scavenging activity}\;(\%)=[\frac{\left({\text{Control}}_{a}-{\text{Sample}}_{a}\right)}{{\text{Control}}_{a}}]\times 100$$ (2)

Where, Control_a_ and Sample_a_ are the absorbance of control (DPPH solution) and samples, respectively. The methanolic DPPH solution (1 mL, 0.3 mmol) was used as a control sample.

#### 2.10.2 ABTS radical scavenging

The ABTS 2,2′-azino-bis(3-ethylbenzothiazoline-6-sulfonic acid) radical scavenging activity was assessed according to the procedure that was described by Sridhar and Charles, (38), with slight alterations. In a stock ABTS solution, equal quantities including those of 7 mM ABTS (aqueous) to 2.45 mM potassium persulfate (aqueous) were placed into a single container. The mixture was allowed to incubate at room temperature in the dark for 12–16 h. This was followed by the addition of 10–120 mm/L ascorbic acid (molecular weight 176.12 g/mol; used as standard) and 5, 10, 20, 40, 80, 100 and 120 μL of the sample and incubated under the aforementioned conditions until a time interval of 10 min was reached. One control contained 1 mL of ABTS solution together with 0.5 mL of distilled water. Absorbance was recorded at 734 nm and thereafter, percentage scavenging activity was determined according to the following Equation 3 (37):


$$\text{Inhibition}\;(\%)=[\frac{\left({\text{Control}}_{a}-{\text{Sample}}_{a}\right)}{{\text{Control}}_{a}}]\times 100$$ (3)

### 2.11 Animal

Male Albino mice, weighing between 30 and 40 g, were used for *in-vivo* study of the topical cream. The animals were housed under standard environmental conditions and experimental protocols approved by the Ethical Committee of Cholistan University of Veterinary and Animal Sciences Bahawalpur (ORIC-328). They were fed with a standard rodent diet and allowed to acclimatize for 2 weeks before the commencement of the experiment.

### 2.12 Diabetes induction

Diabetes was induced by following the method of Pakpahan et al. (39), with minor alteration. Before induction blood sugar level was measured. The tip of mice tail was cleaned with 70% alcohol then piercing with sterile lancet needle. A drop of blood was dripped on a sugar strip to measure the glucose level. Alloxan solution was administered orally (150 mg/kg) as described earlier by Oshkondali et al. (40). The mice were allowed to have access to food and water, as normal. Then stabilized for 7 days after alloxan solution induction. The levels of glucose of ≥250 mg/dL on day seven classify as diabetic (41), and hence qualify individuals to engage in the study of diabetes related experiment.

### 2.13 *In-vivo* experiment

Thirty Albino mice were used for *in-vivo* anti-inflammatory assay. Mice were randomly divided into six groups, with each group consisting of five animals. Mice were anesthetized using a cotton swab that had been dipped in chloroform until the mice were limp (39). The dorsal surface of each mice was shaved and cleaned with a sterile cotton swab. Inflammatory wound was created in this area to with sterile surgical blade, which was then treated according to the group-specific regimen. The progression of wound healing, inflammation reduction, and other relevant parameters was closely monitored.

The groups were categorized as follows: Control group (G_1_), Diabetic model treated with market available cream (G_2_), Diabetic model infected with *Staphylococcus aureus* treated with market cream (G_3_), nanoemulsion incorporated topical cream treated group (G_4_), Diabetic model treated with nanoemulsion incorporated topical cream (G_5_) and Diabetic model infected with *Staphylococcus aureus* treated with nanoemulsion incorporated topical cream (G_6_).

### 2.14 Hematological and histopathological assessment

For the hematological parameters, blood samples were collected in EDTA tubes (42). For histopathological analysis, animals were sacrificed and the skin was excised. The dorsal portion of mice skin was preserved in paraformaldehyde for checking skin tissues. Preserved skin tissues were sectioned using a microtome and stained with acidic dye (eosin) and basic dye (hematoxylin) for visualization under a microscope.

### 2.15 Molecular docking

Molecular docking analysis was performed to assess how eugenol might interact with inflammatory proteins like C-reactive protein (PDB ID: 7PKB) and Interleukin-1β (PDB ID: 9ILB). The structures for these proteins were retrieved from the RCSB PDB database^1^ and the ligand was created from PubChem, Eugenol (PubChem ID: 3314). Each chemical structure chosen to participate in this study was taken from the PubChem database.^2^ Molecular docking was done at the available online-based tool SwissDock provided by the Swiss Institute of Bioinformatics.^3^ The ligand with the maximum binding energy was discarded and the one having the least binding energy thus was regarded as the ideal one to interact with the docking receptor. The visualization process involved presentation in the three-dimensional space through UCSF-Chimera version 1.17.3 (3D and solid surface) and creation of two-dimensional illustrations with the help of BIOVIA Discovery Studio Visualizer (version 4.5) (24).

### 2.16 Statistical analysis

For interpretation of results the datasets collected in the current experiment was statistical analyze using the One-way analysis of variance (ANOVA) using IBM SPSS Statistics, version 22. The data was represented by means and standard error (Means ± SE). The data was presented using (81) (Origin Lab Corporation, MA, USA).

## 3 Results

### 3.1 Preparation and optimization of NE

The CONE was systematically developed and optimized using Box–Behnken response surface design to assess the impact of four independent variables, oil concentration, Tween 80 concentration, sonication time and sonication power on droplet size and PDI. The optimization process revealed that increasing both emulsifier concentration and sonication power significantly reduced droplet size and improved homogeneity. The optimal formulation, comprising 10% (v/v) clove oil, 2.5% (w/v) Tween 80, sonication time of 15 min and 50% sonication power, resulted in a NE with a mean droplet size of approximately 190 nm and a PDI of 0.08. These values reflect a uniform and kinetically stable nanoemulsion system, suitable for enhanced topical delivery applications.

### 3.2 Gas chromatography–mass spectrometry (GC/MS) analysis

**Figure 1:**
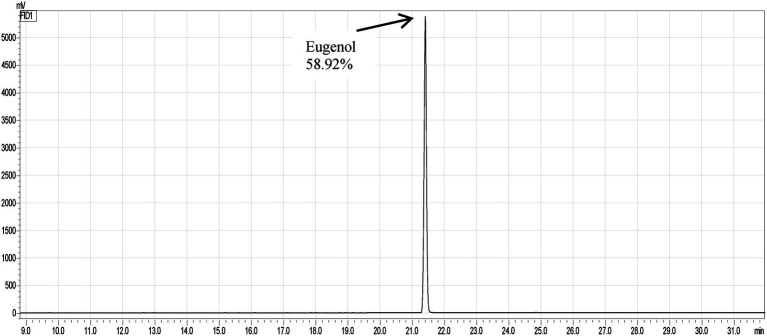
Gas Chromatography–Mass Spectrometry (GC/MS) analysis of clove oil.

The gas chromatographic analysis presented the retention time and peak areas of the major compound in CEO. The major compound is eugenol (58.92%). The retention time (21.30 min) and peak area of eugenol 892,342 au (arbitrary units) are shown in Figure 1.

### 3.3 Transmission electron microscopy (TEM)

**Figure 2:**
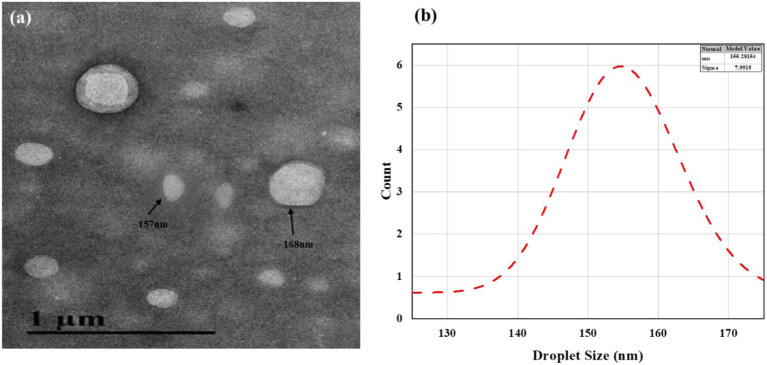
**(a)** Transmission Electron Microscope image of CONE; **(b)** Normal Distribution Model of Droplet size visualized by TEM micrograph.

The NE particles morphology and size of the optimized CONE were examined using TEM. It was indicated in Figure 2 that the core of each particle is lighter than the periphery, confirming the oil-in-water (O/W) nature of the NEs. The droplets were spherical, with a smooth surface and uniform dispersion. The droplets appeared well-dispersed without significant aggregation, suggesting good colloidal stability. The nanoemulsion droplet size ranged from 20 to 200 nm, with a mean value of 155.28 ± 7.99 nm as depicted in Figure 2b.

### 3.4 Entrapment efficiency

The optimal CONE was used to measure their entrapment efficiency by UV-spectrophotometry at the wavelength of 280 nm. CONE had an average entrapment efficiency of about 94.54% because of an effective formulation procedure and controllable oil dispersion in the NE matrix.

### 3.5 Properties of topical cream

Creams containing CONE were visually confirmed after being kept at heating–cooling conditions. The cream color and smell remained unchanged after the heating–cooling test compared with those before. The pH of cream containing NE before and after the heating–cooling test was found to be in the range of 6.5–8.0. The formulations NE cream immobilized kept pH values stable with few increases after the heating and cooling process. The resultant cream was stable with no separation. The properties of cream are shown in Table 1.

**Table 1:** Physical properties of cream embedded with clove oil nanoemulsion.

Parameter	0.5 mL		0.75 mL		1 mL		1.5 mL	
	Before	After	Before	After	Before	After	Before	After
Appearance	Semisolid	Semisolid	Semisolid	Semisolid	Semisolid	Semisolid	Semisolid	Semisolid
Color	Cloudy white	No change	Cloudy white	No change	Cloudy white	No change	Cloudy white	No change
Smell	Characteristic	Characteristic	Characteristic	Characteristic	Characteristic	Characteristic	Characteristic	Characteristic
Texture	Smooth and consistent	Smooth and consistent	Smooth and consistent	Smooth and consistent	Smooth and consistent	Smooth and consistent	Smooth and consistent	Smooth and consistent
pH value	6.85	6.94	7.12	7.16	7.37	7.41	7.57	7.61
Separation	No	No	No	No	No	No	No	No
Stability	Stable	Stable	Stable	Stable	Stable	Stable	Stable	Stable

Various batches prepared were subjected to evaluation of organoleptic characters such as colour, odor, uniformity, physical stability, pH and spreadability. The most optimized formula was chosen according to the above parameters analysis and the release profile of the nanoemulsion. The optimized and stable formulation was then tested.

### 3.6 Sustain release of nanoemulsion in cream

**Figure 3:**
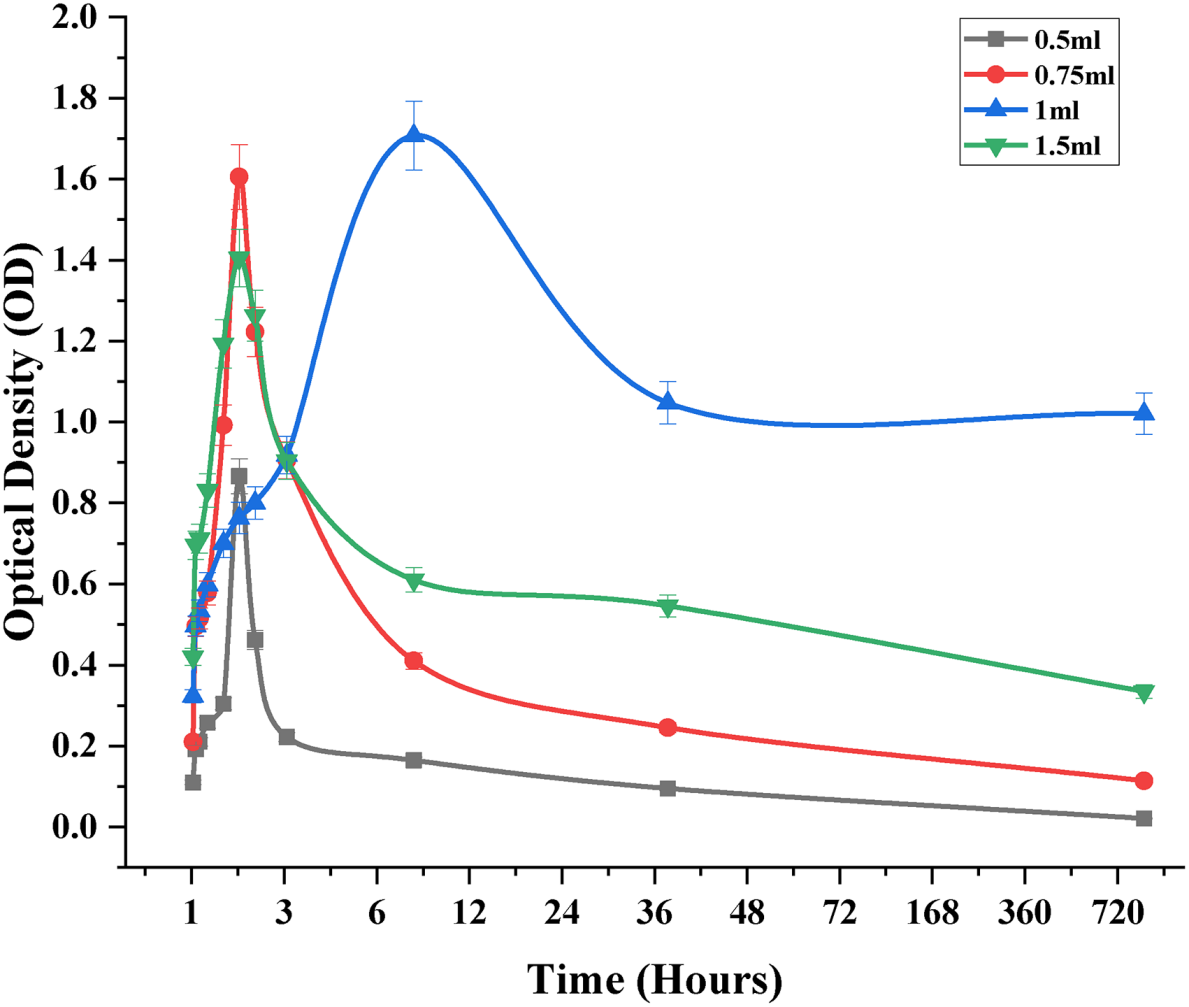
Drug release trend of clove oil nanoemulsion from cream after different time intervals.

*In-vitro* release studies of CONE incorporated topical cream formulations were performed using UV–Vis spectrophotometry. The release kinetics exhibited a biphasic trend with an initial burst release, followed by a sustained release pattern up to 72 h. Among all formulations (0.5, 0.75, 1 and 1.5 mL of CONE), only 1 mL formulation demonstrated the most favorable release behavior, balancing rapid initial diffusion with prolonged drug availability (Figure 3).

The initial burst phase observed across all formulations is attributed to the rapid diffusion of surface-associated CEO molecules. However, 1 mL formulation exhibited a more controlled release trajectory, indicative of efficient entrapment and regulated diffusion from the cream matrix. In contrast, the 0.5 and 0.75 mL formulations displayed a sharp decline in drug release after the initial phase, suggesting premature depletion of the active compound and limited sustained release capacity. The 1.5 mL formulation showed a delayed onset of release with extended retention, potentially due to increased formulation viscosity or enhanced encapsulation hindering immediate diffusion.

At 24 and 48 h, 1 mL CONE cream maintained a steady release profile, whereas lower concentrations exhibited marked depletion. By 72 h, the 0.5 and 0.75 mL formulations approached complete release, while 1 mL formulation retained a therapeutically relevant concentration, confirming its superior sustained release characteristics. Although the 1.5 mL formulation also demonstrated prolonged retention, its lower initial release and delayed kinetics may necessitate further optimization to balance therapeutic efficacy and diffusion control. The release data kinetic modelling showed that the Higuchi model was best able to describe the general release pattern which established diffusion-controlled mechanism. These findings suggest that 1 mL CONE concentration offers an optimal release profile for topical delivery, providing both immediate therapeutic action and extended drug retention, which are critical for effective anti-inflammatory outcomes.

### 3.7 Morphological study of cream

**Figure 4:**
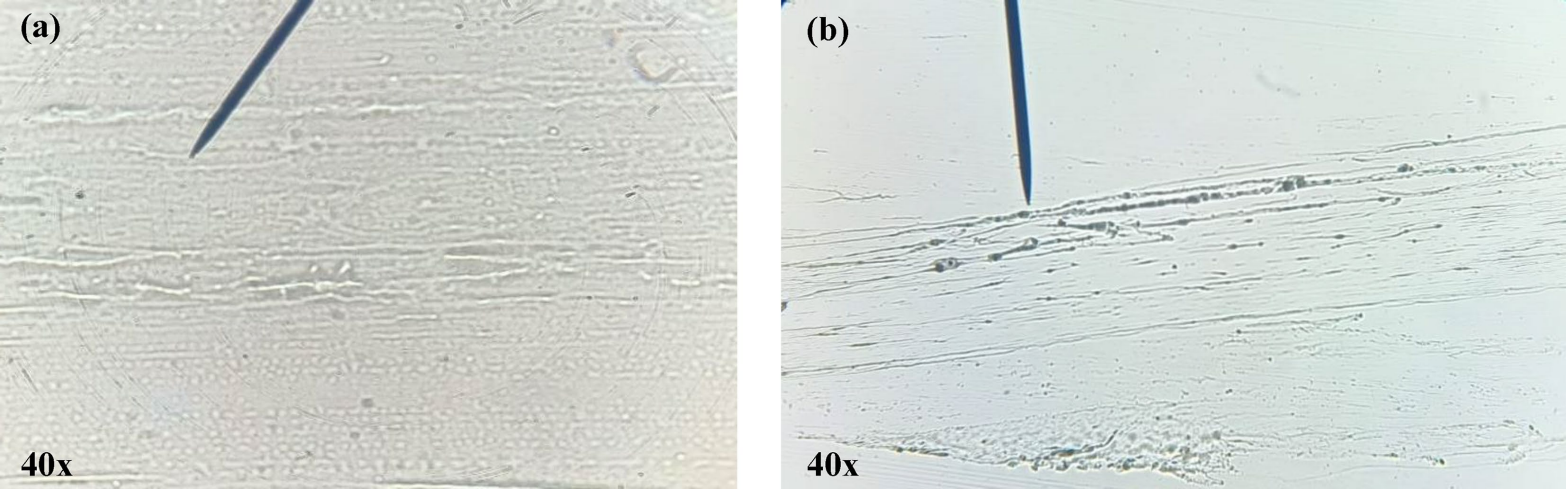
Microscopic examination of the polymer and CONE incorporated cream. **(a)** Carboxymethyl cellulose polymer, **(b)** CONE loaded cream.

The morphological characteristics of the cream and carboxymethyl cellulose (CMC) were evaluated using compound microscopy, as illustrated in Figure 4. The CMC matrix exhibited a fibrous, thread-like morphology with irregular striations, indicative of its polymeric nature and its role in imparting structural integrity to the formulation (Figure 4a). Uniformly distributed circular formations were also observed, suggesting consistent dispersion. In contrast, the CONE-based cream displayed a morphology consistent with an O/W emulsion, characterized by numerous fine oil globules dispersed within a continuous aqueous phase as presented in Figure 4b. The absence of phase separation under microscopic observation confirms successful encapsulation of the oil phase and the formation of a stable NE-based cream system.

### 3.8 Antifungal activity

**Figure 5:**
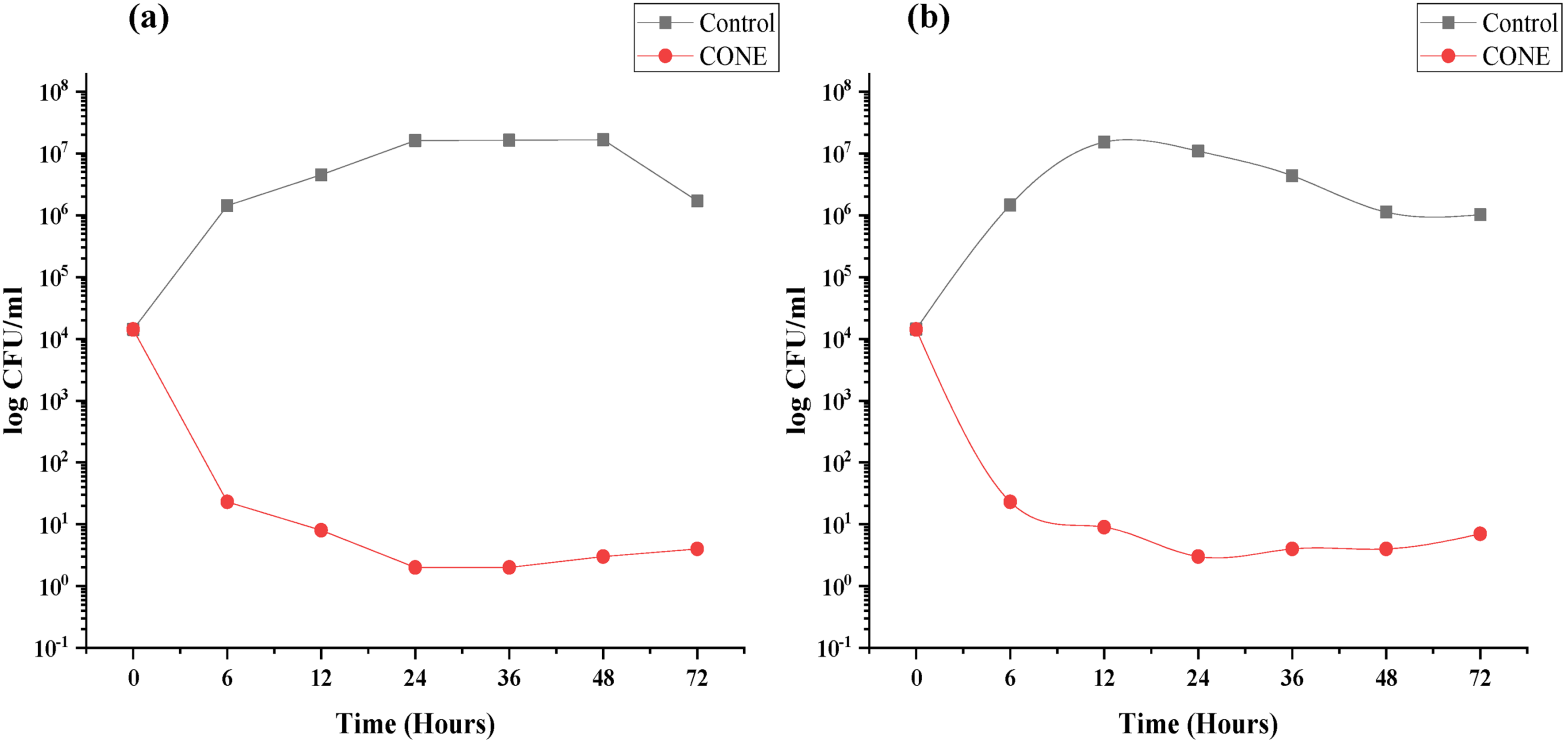
Time–kill plot for clove oil nanoemulsion (CONE). **(a)** *Penicillium italicum*; **(b)** *Aspergillus niger*.

The promising potential of CONE was also highlighted as an effective antifungal agent. The antifungal assay was performed against *P. italicum* and *A. niger*. CONE strongly inhibits the growth of fungal strains as compared to their controls. Both the fungal strains showed almost the same trend and MIC (120 μL). It was observed that CONE strongly inhibits *P. italicum* as compared to *A. niger*. Figure 5 revealed that the fungal growth was reduced to almost 10^1^ CFU/mL during the first 12 h and no significant growth was observed. While in the control group, the growth was significantly increased to 10^6^ CFU/mL even after 6 h in the case of *A. niger* and it rose to 10^7^ CFU/mL during 12 h. Later, it reduced to 10^6^ CFU/mL till 72 h. While in the case of *P. italicum* control, maximum growth (10^7^ CFU/mL) was observed after 24 h.

### 3.9 Antioxidant activity of CONE

The antioxidant potential of CEO and CONE was examined using the DPPH and ABTS radical scavenging assays. A dose-dependent increase in scavenging activity was observed for both CEO and CONE across a concentration range of 10 μL to 100 μL. However, the CONE consistently exhibited significantly higher scavenging efficiency compared to the pure CEO across all tested concentrations.

**Figure 6:**
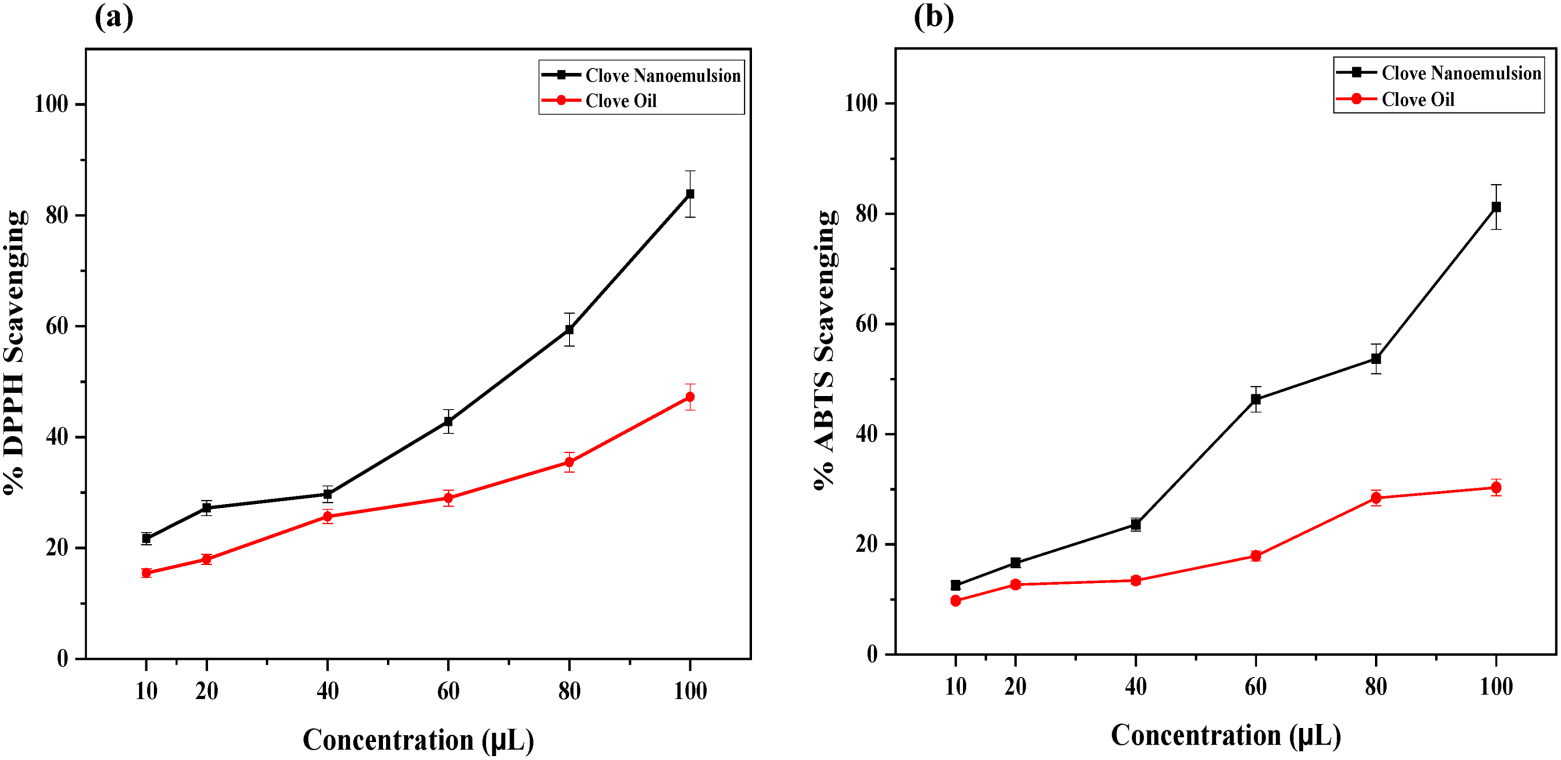
Antioxidant activities of clove essential oil and clove oil nanoemulsion **(a)** DPPH **(b)** ABTS. DPPH, (1,1-diphenyl picryl hydrazyl); ABTS, [2,2-azino-bis (3-ethylbenzothiazoline-6-sulfonic acid)].

The results demonstrated that the CONE exhibited significantly enhanced antioxidant activity compared to the pure clove oil at all tested concentrations. At the highest concentration (100 μL), the NE achieved a maximum scavenging percentage of approximately 84%, while the unencapsulated clove oil showed about 47% scavenging activity as presented in Figure 6a. This enhancement may be attributed to the increased surface area, improved dispersion, and greater bioavailability of the active constituents in the nano-emulsified form. These findings suggest that nano-emulsification of CEO not only preserves but significantly improves its antioxidant efficacy, making it a promising candidate for therapeutic and cosmetic formulations where oxidative stress plays a central role. The result represented that at the highest concentration tested (100 μL), the CONE achieved a maximum scavenging activity of approximately 81%, while the corresponding value for CO was observed at around 30.3% (Figure 6b). This marked enhancement in antioxidant activity in the NE formulation may be attributed to the reduction of droplet size, which facilitates improved dispersion and solubility of active constituents such as eugenol. Additionally, the increased surface area of the NE droplets likely promotes more effective interaction with the ABTS radicals. *In-vivo* anti-inflammatory analysis.

**Figure 7:**
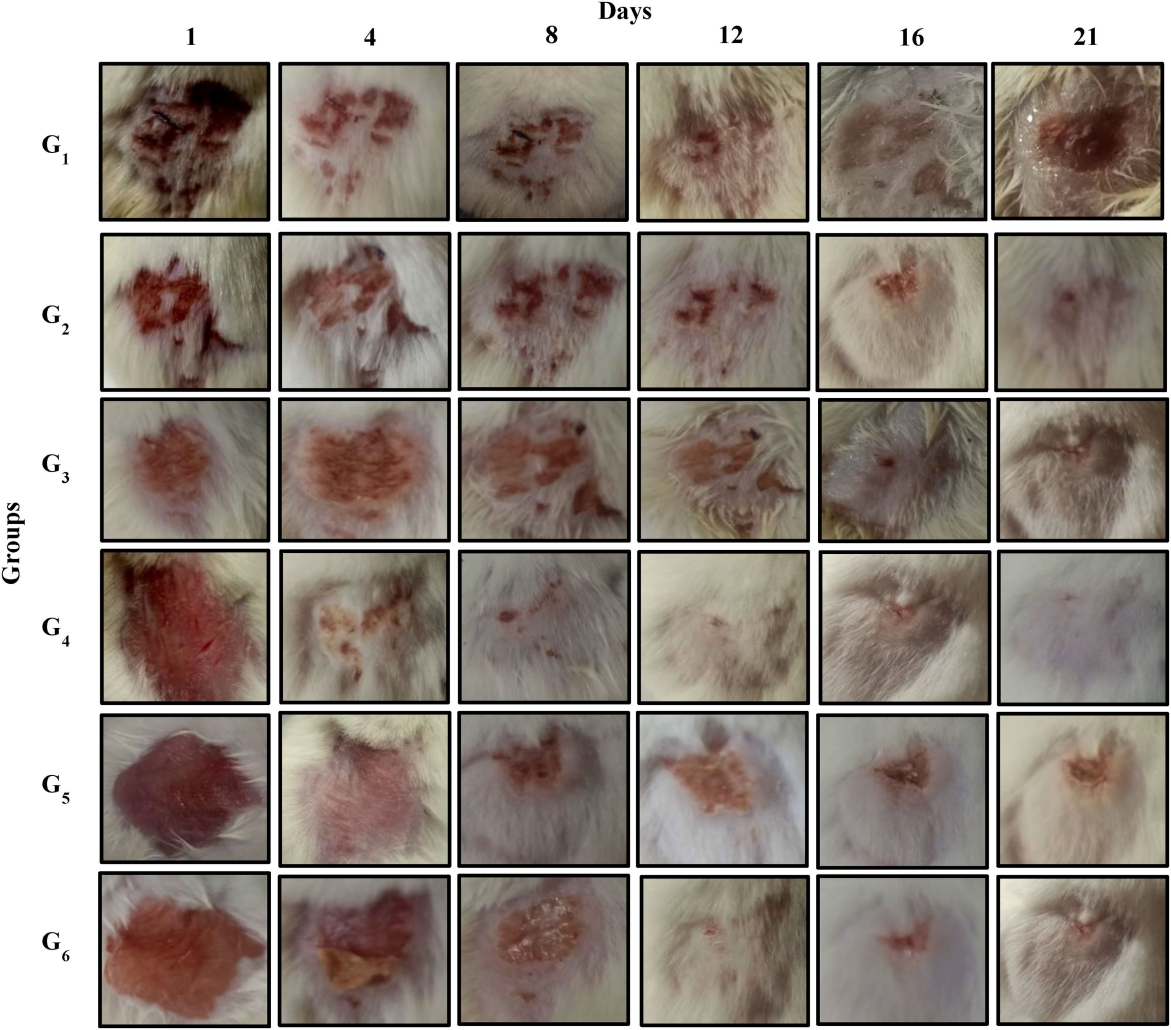
*In-vitro* trail images of inflammatory mice models at different days. Control group **(G**_**1**_**)**; diabetic model treated with market available cream (dicloran gel) **(G**_**2**_**)**; diabetic model infected with *S. aureus* treated with market cream **(G**_**3**_**)**; CONE incorporated topical cream treated group **(G**_**4**_**)**, diabetic model treated with CONE incorporated cream **(G**_**5**_**)**; diabetic model infected with *S. aureus* treated with CONE incorporated cream **(G**_**6**_**)**.

The experimental study assessed the sequential progression of cutaneous inflammation in six groups (G_1_–G_6_) at multiple time points (1, 4, 8, 12, 16, and 21 days). On day 1, all groups exhibited significant inflammation, evidenced by pronounced redness, swelling, and skin lesions. By day 4, groups treated with CONE-based cream (G_4_ and G_5_) began to show improvement, with reduced redness and smaller lesion areas, while the control group and the group treated with a marketed dicloran gel (G_2_ and G_3_) continued to display persistent inflammation; G_6_ showed moderate improvement, consistent with visual data. From day 8 onward, groups receiving CONE-based cream demonstrated marked healing, with significant reduction in redness and lesion size, whereas the control group remained largely unchanged, exhibiting ongoing severe inflammation. Groups G_2_ and G_6_ showed gradual but less pronounced improvement. After day 21, animals in G_4_ and G_5_ exhibited almost complete skin healing with only faint residual marks, while the control group showed delayed recovery with lingering redness. Groups with severe inflammation due to *S. aureus* addition (G_3_ and G_6_) achieved moderate healing but retained evidence of tissue damage as presented in Figure 7.

### 3.10 Hematological analysis

Hematological profiling was performed to evaluate the systemic effects of CONE-based topical cream across six experimental groups: G_1_ (control), G_2_ (diabetic model + market cream), G_3_ (diabetic + *Staphylococcus aureus* + market cream), G_4_ (CONE cream only), G_5_ (diabetic + CONE cream), and G_6_ (diabetic + *S. aureus* + CONE cream). As summarized in Table 2, significant alterations in hematological parameters were observed among the groups.

**Table 2:** Hematological parameters of different experimental groups (G1–G6).

Parameters	G_1_	G_2_	G_3_	G_4_	G_5_	G_6_
WBC	8.35 ± 0.05^a^	8.33 ± 0.02^a^	8.31 ± 0.02^a^	5.15 ± 0.04^c^	6.28 ± 0.02^b^	8.27 ± 0.02^a^
LYM%	87.37 ± 0.02^a^	87.28 ± 0.03^a^	87.17 ± 0.05^a^	72.83 ± 0.02^b^	73.03 ± 0.06^b^	87.15 ± 0.01^a^
MID%	8.29 ± 0.03^a^	8.35 ± 0.03^a^	7.24 ± 0.03^b^	6.59 ± 0.01^c^	7.25 ± 0.04^b^	6.62 ± 0.03^c^
GR%	18.54 ± 0.03^a^	18.47 ± 0.03^a^	17.54 ± 0.04^b^	9.24 ± 0.03^d^	17.56 ± 0.01^b^	10.33 ± 0.02^c^
RBC	4.35 ± 0.04^d^	4.38 ± 0.01^d^	4.82 ± 0.03^c^	7.30 ± 0.26^a^	5.88 ± 0.01^b^	4.83 ± 0.03^c^
HGB	65.35 ± 0.04^e^	95.32 ± 0.02^c^	95.35 ± 0.04^c^	135.22 ± 0.01^a^	99.54 ± 0.02^b^	89.35 ± 0.04^d^
HCT	37.27 ± 0.04^d^	37.54 ± 0.03^c^	38.36 ± 0.02^b^	38.41 ± 0.03^b^	45.30 ± 0.01^a^	38.36 ± 0.02^b^
MCV	53.72 ± 0.02^d^	53.69 ± 0.01^d^	54.48 ± 0.03^b^	61.09 ± 0.04^a^	54.45 ± 0.03^bc^	54.40 ± 0.02^c^
MCH	15.54 ± 0.04^d^	18.34 ± 0.04^c^	15.36 ± 0.02^e^	20.10 ± 0.02^a^	18.37 ± 0.03^bc^	18.43 ± 0.04^b^
MCHC	265.73 ± 0.03^d^	267.17 ± 0.04^c^	265.14 ± 0.03^e^	302.45 ± 0.03^a^	278.12 ± 0.02^b^	267.13 ± 0.01^c^
PLT	812.16 ± 0.04^a^	812.20 ± 0.02^a^	723.72 ± 0.02^b^	681.27 ± 0.02^d^	684.45 ± 0.03^c^	723.68 ± 0.03^b^
MPV	10.34 ± 0.05^a^	9.81 ± 0.02^bc^	9.77 ± 0.02^cd^	6.42 ± 0.03^e^	9.86 ± 0.02^b^	9.73 ± 0.02^d^
PCT	0.45 ± 0.03^a^	0.36 ± 0.04^d^	0.42 ± 0.02^ab^	0.24 ± 0.02^e^	0.38 ± 0.01^cd^	0.42 ± 0.02^ab^
PDW	18.9 ± 0.04^a^	18.6 ± 0.03^b^	18.9 ± 0.02^a^	15.9 ± 0.02^d^	16.4 ± 0.03^c^	18.6 ± 0.03^b^

Different superscript letters (a–e) within a row indicate statistically significant differences (*p* < 0.05).

WBC, white blood cell count; LYM, lymphocyte; MID, mid-sized cell; GR, granulocyte; RBC, red blood cell count; HGB, hemoglobin; HCT, hematocrit; MCV, mean corpuscular volume; MCH, mean corpuscular hemoglobin; MCHC, mean corpuscular hemoglobin concentration; PLT, platelet; MPV, mean platelet volume; PCT, plateletcrit; PDW, platelet distribution width.

White blood cell (WBC) counts were notably reduced in G_4_ (5.15 ± 0.04) and G_5_ (6.28 ± 0.02) compared to the other groups, potentially indicating an immunomodulatory or suppressive effect of the CONE-based formulation. A similar trend was observed in lymphocyte percentages, which were significantly lower in G_4_ (72.83 ± 0.02) and G_5_ (73.03 ± 0.06), suggesting modulation of immune cell populations. The monocyte and eosinophil (MID) counts were also decreased in G_4_, G_5_, G_6_, and G_3_, implying altered leukocyte differentiation. Granulocyte (GR) percentages were significantly reduced in G_4_ (9.24 ± 0.03) and G_6_ (10.33 ± 0.02), further supporting immunological modulation by the nanoemulsion-based cream treatment.

Erythrocytic indices reflected substantial variation across groups. Red blood cell (RBC) counts were significantly elevated in G_4_ (7.30 ± 0.26) and G_5_ (5.88 ± 0.01), suggesting enhanced erythropoiesis. Hemoglobin (HGB) concentrations were also markedly increased in G_4_ (135.22 ± 0.01) and G_5_ (99.54 ± 0.02), while G_1_ exhibited the lowest levels (65.35 ± 0.04). Hematocrit (HCT) was highest in G_5_ (45.30 ± 0.01), indicating improved oxygen-carrying capacity. Mean corpuscular volume, mean corpuscular hemoglobin and mean corpuscular hemoglobin concentration were all significantly elevated in animals treated with nanoemulsion incorporated cream (G_4_), further supporting improved erythrocyte quality and function following treatment.

Platelet indices also exhibited statistically significant differences. PLT was highest in G_1_ and G_2_, but significantly reduced in G_4_ (681.27 ± 0.02) and G_5_ (684.45 ± 0.03), possibly reflecting infection-related thrombocytopenia or platelet consumption. Mean platelet volume, plateletcrit and platelet distribution width (PDW) were significantly reduced in G_4_, indicating altered platelet morphology and activation status. Collectively, these findings suggest that topical application of CONE modulates hematological parameters, including immune cell counts, erythropoietic markers and platelet indices. The observed variations indicate potential immunomodulatory and hematopoietic effects, warranting further mechanistic investigation.

### 3.11 C-reactive protein

**Figure 8:**
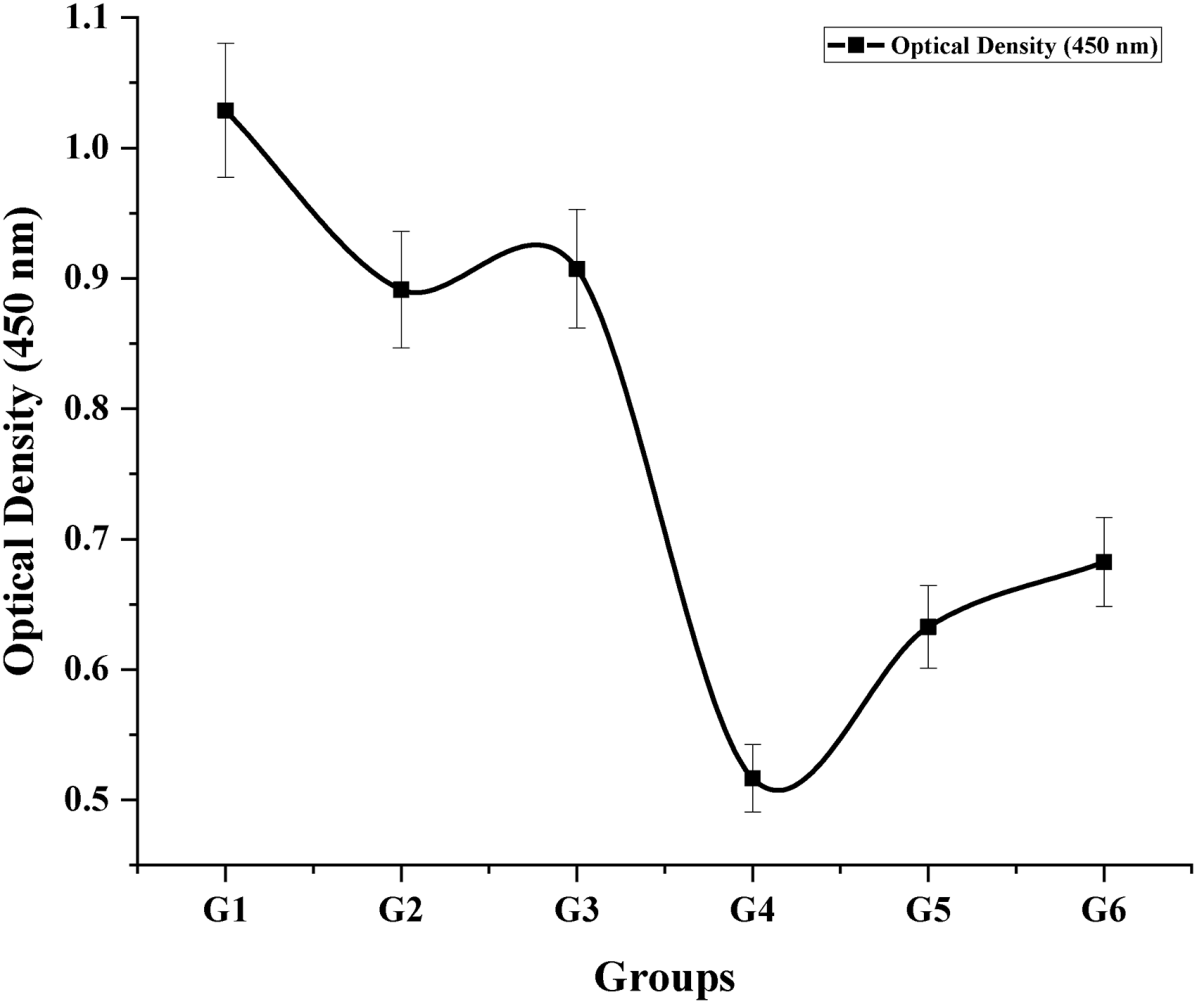
Optical density of C-reactive protein from serum of different mice groups.

The concentration of C-reactive protein (CRP), a key acute-phase inflammatory biomarker, was quantitatively evaluated in mouse serum using a UV–Vis spectrophotometer at 450 nm. Optical density (OD) values corresponding to CRP levels were recorded across all experimental groups (Figure 8). A normal value 0.435 ± 0.01 was recorded for healthy mice. Significantly elevated CRP levels were detected in inflammatory-induced and *Staphylococcus aureus*-infected groups, indicative of systemic inflammation. Interestingly, the highest CRP value was observed in the untreated control group, suggesting active inflammatory processes. Conversely, mice treated with the CONE-based cream (G_4_) exhibited a substantial reduction in CRP levels, closely approaching baseline values. This reduction reflects the anti-inflammatory potential of the nanoemulsion formulation and supports its efficacy in attenuating systemic inflammatory responses *in-vivo*.

### 3.12 Histopathological analysis

**Figure 9:**
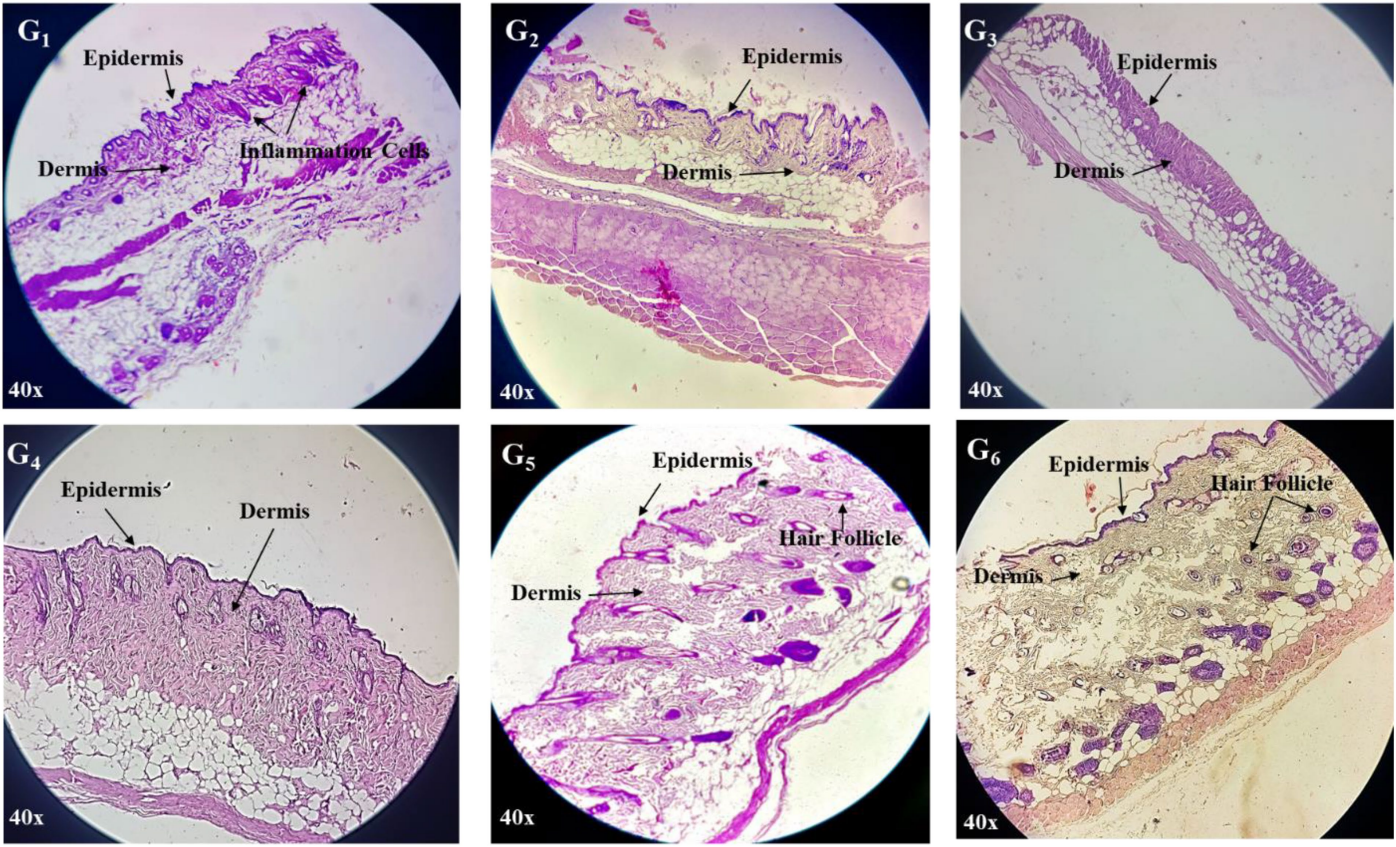
Histopathological examination of mice skin tissues. Control group **(G**_**1**_**)**; diabetic model treated with market available cream (dicloran gel) **(G**_**2**_**)**; diabetic model infected with *S. aureus* treated with market cream **(G**_**3**_**)**; CONE incorporated topical cream treated group **(G**_**4**_**)**, diabetic model treated with CONE incorporated cream **(G**_**5**_**)**; diabetic model infected with *S. aureus* treated with CONE incorporated cream **(G**_**6**_**)**.

After completion of the anti-inflammatory experiment, skin tissues from all groups were collected and subjected to histopathological examination using hematoxylin and eosin (H&E) staining. The histological analysis aimed to evaluate the extent of epidermal and dermal recovery, collagen organization, inflammatory infiltration, and tissue architecture preservation (Figure 9). Histological evaluation revealed pronounced epidermal and dermal disruption, characterized by thickened and dense infiltration of inflammatory cells in the control group (G_1_). The diabetic group (G_2_) treated with dicloran gel (marketed) exhibited partial epidermal restoration with thin, disorganized layers and moderate collagen disarray, along with persistent inflammatory infiltration. Hair follicles appeared sparse and atrophic.

The diabetic group infected with *S. aureus* and treated with market cream (G_3_) displayed the most severe histopathological alterations, including epidermal erosion, dermal edema, extensive inflammatory infiltration, and collagen degradation. There was a high presence of polymorphonuclear cells, indicating acute inflammation and a lack of proper tissue healing. On the other hand, G_4_: the group treated with CONE-based cream, showing significant improvement in histology. The epidermis showed good continuity and stratification, and the dermis revealed dense collagen bundles with few inflammatory infiltrates. The hair follicle was preserved, indicating that the formulation may have an initial healing potential. Diabetic group treated with CONE cream (G_5_) showed marked epidermal hyperplasia, regular arrangement of cells, and senile cell presence with mild degree of dermal architecture. The hair follicles were excessive and morphologically normal. Likewise, in G_6_ (diabetic + *S. aureus* + CONE cream), significant improvement in skin architecture was observed with re-established epidermal continuity, organized collagen deposition, and partial preservation of dermal appendages with mild remnant inflammation. Collectively, these findings confirm the therapeutic potential of the CONE-based cream in promoting epidermal and dermal regeneration, suppressing inflammation, and preserving skin integrity, even under diabetic and infectious conditions.

### 3.13 Molecular docking

**Figure 10:**
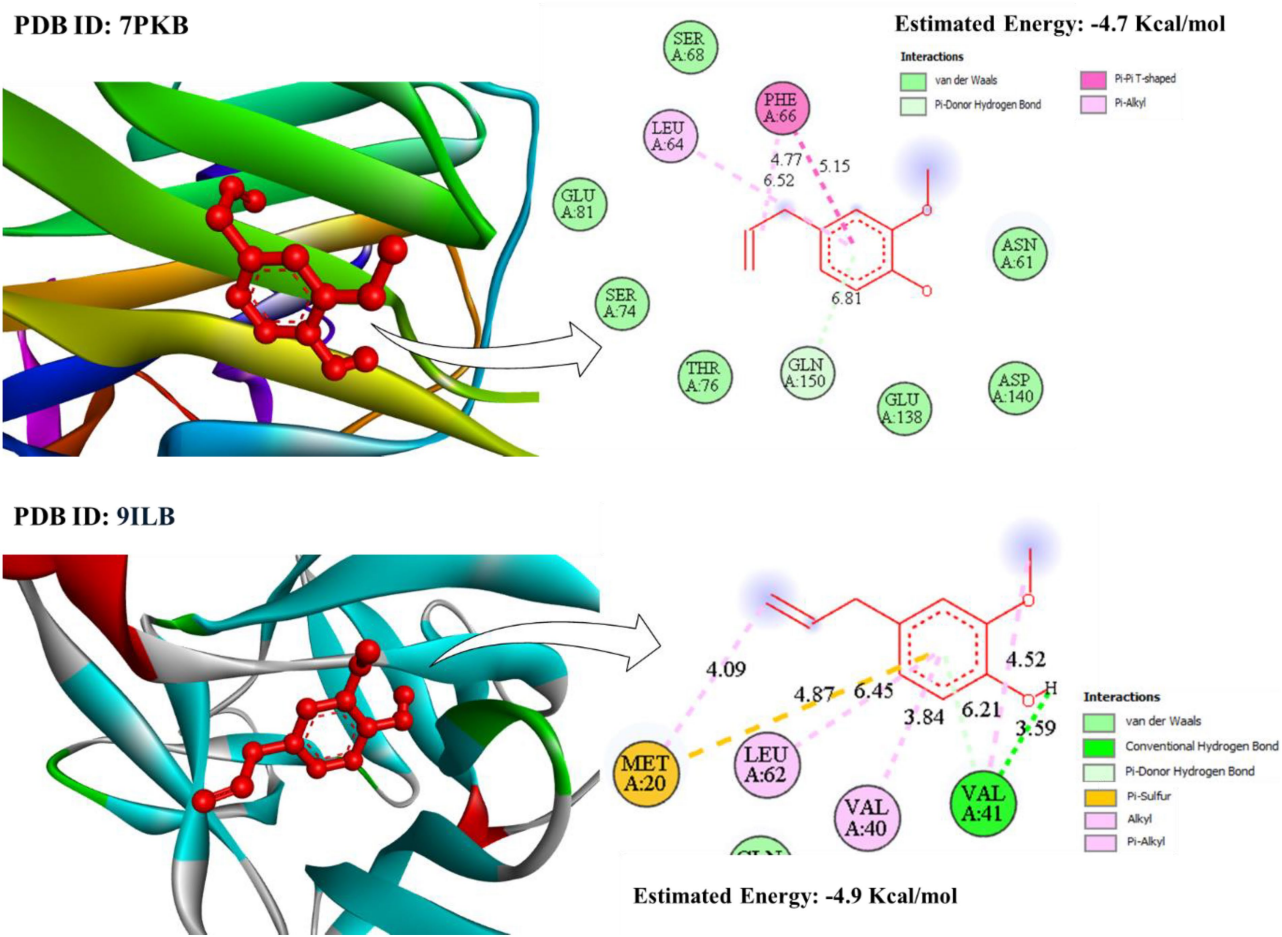
Molecular interactions between of eugenol and inflammatory proteins. PKB, C-reactive protein; 9ILB, Interleukin‑1β.

The molecular docking analysis of eugenol with inflammatory protein targets revealed significant binding affinities and interaction patterns supporting its anti-inflammatory potential. It was observed that eugenol exhibited binding energies of approximately −4.7 kcal/mol with C-reactive protein (7PKB) and −4.9 kcal/mol with Interleukin-1β (9ILB). In case of CRP, eugenol engages in a *π*–π T-shaped interaction with PHE66 and π-alkyl interaction with LEU64, at distances of 6.52 Å and 4.77 Å, respectively. Stabilization is further supported by van der Waals contacts involving SER68, SER74, THR76, GLU81 and GLN150, anchoring the aromatic ring deep within the concave pocket reminiscent of the phosphocholine-binding site in CRP (Figure 10).

In Interleukin-1β (IL-1β; PDB ID: 9ILB), eugenol similarly achieved a favorable docked pose. A conventional hydrogen bond is formed with VAL41 (3.59 Å), accompanied by π-alkyl and alkyl contacts with LEU62 and VAL40, respectively. Notably, a distinct π-sulfur interaction with MET20 and additional π-alkyl interactions at distances between ~4.09 and 4.87 Å reinforce the binding. These combined interactions indicate a robust multi-mode binding paradigm within the IL-1β hydrophobic cavity (Figure 10).

## 4 Discussion

### 4.1 Optimization of NE

The CONE was optimized using response surface methodology, focusing on the effects of oil concentration, Tween 80, sonication time, and power on droplet size and PDI. The optimized formulation (10% v/v oil, 2.5% w/v Tween 80, 15 min sonication, 50% power) yielded the smallest droplet size (190 nm) and lowest PDI (0.08), indicating high stability and uniformity. Droplet sizes under 300 nm are known to enhance drug permeation (43), and the surfactant likely stabilized droplets by forming a compact interfacial film (44). Longer sonication time further reduced droplet size and PDI, likely due to enhanced cavitation effects, as supported by Ahmed et al. (45). When the PDI is smaller than 0.3, the nanoemulsion system has a narrow size distribution (46). The optimized NE showed PDI < 0.1, confirming narrow size distribution, which is suitable for topical delivery.

### 4.2 Entrapment efficiency

The present study demonstrated a high EE (94%) for CONE, aligning with previous reports on EO-based NE, where effective emulsification and optimized surfactant concentrations contribute to enhanced drug loading (47). The EE is primarily dependent on the solubility of the active, its compatibility with the constituents of the formulation. The poor aqueous solubility of CEO components might play in favor of encapsulation, which is further facilitated in the presence of surfactants and co-surfactants (48). Whereas those with very high levels of surfactants, such as emulsions with 74% Tween 80, can result in low entrapments, either by micellar solubilization, as has been reported in a few NEs and nano-emulgel (49).

### 4.3 Properties of topical cream

The organoleptic properties and pH of the prepared formulations are presented in Table 1. No evidence for phase separation or inhomogeneity was noticed, which may be ascribed to the utilization of nonionic surfactants characterized by stability against ionic strength and pH adjustments (50). During the stability study, the preparations were microbiologically stable and had no variation in odor, attributable possibly to the emulsion’s natural antioxidants and preservatives. The pH value is an important part of the stability and dermatological compatibility of topical formulations (51). The pH values of the prepared cream formulations were all in the compatible pH range of skin (4–7) (52), establishing that the products are suitable for topical application without any irritation.

### 4.4 Sustain release

*In-vitro* release studies are important preliminary investigations to gain knowledge on the *in-vivo* behavior of topical preparations, like release kinetics and mechanism. The *in-vitro* release pattern of CONE containing cream exhibited a typical biphasic one, as previously reported (53). Biphases kinetic profiles are commonly reported in lipid-based nanocarriers and nanoemulsions, where a burst phase can be observed at the onset of the therapy to ensure fast therapeutic delivery, and a further sustained phase to provide delayed availability to the drug (54). Droplet size, surfactant interfacial thickness, and drug-lipid interactions are some of the parameters that mediate the diffusion-controlled release stage. In the current study, the consistent release data with the Higuchi model proves that diffusion by the nanoemulsion-based matrix is the major force in release. This effect has been already described in polymeric and lipid nanoparticles, in particular with lipophilic and poorly water-soluble drugs, in which the matrix-controlled systems surpass traditional formulations to support long activity (55).

An initial burst release was seen at all the tested concentrations which resulted from the quick diffusion of clove oil, adsorbed on the surface of NE droplets. This was succeeded by the sustained release stage, controlled by the slowest release of drug due to diffusion from the lipid core that worked as a reservoir system. Such biphasic release profile can provide dual benefits of immediate availability of therapeutic moiety as well as a sustained effect, which is consistent with previous studies on nanoemulsion topical preparations (56).

Cream incorporated with 1 mL of CONE showed the best released pattern, although a burst release was observed after 72 h. Low concentrations CONE formulations (0.5 and 0.75 mL) which had a limited encapsulation, showed faster and burst release. On the other side the higher concentration of CONE (1.5 mL) showed irregular release pattern due to droplet agglomeration or saturation effects. The sustained and controlled release behavior of the 1 mL formulation indicates its suitability for extended dermal therapeutic efficacy (57, 58). This release mechanism that is controlled by diffusion ensures long retention of the active compound to the site of application; thus, therapeutic effectiveness is improved and offers less frequent reapplication. Comparable sustained release of eugenol loaded nanoemulsions have already been described by Ahmad et al. (59), explained by small, homogeneous droplets. Likewise, Aldeeb et al. (56) observed that an optimized release was obtained from citronellol nanoemulgels concerning the concentrations of the formulation, suggesting the importance of the parameters of formulation in controlling skin absorption. Taken together, these results establish the 1 mL CONE composition as being optimal for immediate and extended drug delivery.

### 4.5 Antifungal

CONE showed strong antifungal activity against *Penicillium italicum* and *Aspergillus niger*, causing significant inhibition of their growth compared to the control. The MIC values were similar for both fungal strains, demonstrating the broad-spectrum antifungal activity. Those results are consistent with those of Tomić et al. (60), who also documented comparable antifungal activities of essential oil nanoemulsion on *Cladosporium cladosporoides, Aspergillus fumigatus* and *Penicillium chrysogenum.* Fungal strains (*P. italicum* and *A. niger*) were chosen due to their importance as typical postharvest and opportunistic pathogens. In addition, the time-kill kinetics pattern indicated that CONE showed a quick and potent inhibition activity against *P. italicum*, which is acquiring fungicidal activity rather than fungistatic activity. This is also in agreement with Sim et al. (61), who highlighted the practical significance of the time-kill assays on *in-vitro* fungicidal potency of plant extracts at the MIC values.

### 4.6 Antioxidant assays

Nanoencapsulation has been confirmed by several studies to significantly enhance the antioxidant activity of natural products (62). This enhancement is achieved by improving the stability, bioavailability, and dispersibility of active components, protecting them from environmental degradation, and facilitating a greater interaction with free radicals due to the increased surface area at the nanoscale (63). In the present investigation, the antioxidant activity of CONE was tested using DPPH and ABTS radical scavenging. The free radical scavenging ability of CONE showed a dose-dependent increase and maximum DPPH inhibition (~84%) was observed at 100 μL, as compared to bulk clove oil 47%. These results were in agreement with those of Nagaraju et al. (37) also revealed 80% of DPPH radical scavenging activity at 100 μg/mL for clove oil nano emulsion. ABTS assay also showed 81% inhibition by CONE versus 30.3% by clove oil. On the whole, increased radical-scavenging activity of CONE suggests that nanoencapsulation greatly enhances the antioxidant activity of clove oil.

### 4.7 *In-vivo* anti-inflammatory activity

The *in-vivo* study reported that the topical creams incorporated with CONE significantly reduced skin inflammation and promoted wound healing in mice, even in diabetic and infected conditions. During the 21-days treatment period, the groups treated with CONE-based cream (G_4_ and G_5_) presented significant decreases in inflammatory area and were then in nearly complete remission. These results are better than a non-treated group and dicloran gel ones, confirming the higher efficiency of NE base. Another anti-inflammatory and regenerative aspect of clove oil was reported by Banerjee et al. (64), who detected decreased paw edema and increased wound closure in rats. Likewise, El-Zahaby et al. (11) found that the wound closure rate was enhanced by clove oil encapsulated nanostructured liquid crystals and showed the same result on wound healing in infected burn wounds. Mohamed et al. (65) demonstrated decreased pro-inflammatory cytokines and oxidative stress in clove oil nanoemulsion-treated mice.

Hematological examination showed raised RBC and HGB, while lowered WBC and platelet counts in CONE-treated groups. This reduction may be attributed to the anti-inflammatory and antioxidant actions of eugenol, the principal bioactive component of clove oil, which has been reported to inhibit the release of pro-inflammatory cytokines such as TNF-α, IL-1β, and IL-6 (66). By attenuating these mediators, eugenol can reduce leukocyte recruitment and proliferation, leading to stabilization of WBC levels during inflammation. Furthermore, the nanoemulsion delivery system enhances cutaneous penetration while minimizing systemic immune activation (67), thereby suppressing peripheral leukocytosis often seen in diabetic or infected models. Together, these effects may explain the lower WBC counts in G_4_ and G_5_ groups. Elgharib et al. (68), CEO restored hematological indices towards normal in the rats treated with cadmium chloride and supported its protective action against systemic toxicity. Similarly, Pandey et al. (10) stress that it is the context-dependent hematological effects of clove oil nanoemulsions that should be considered and further studied.

There were statistically significant differences in platelet indices in the experimental groups. The highest number of platelets (PLT) was seen in this case of G_1_ and G_2,_ as compared to G_4_ and G_5,_ which had significantly lower numbers. The platelet count decrease observed can be mechanistically explained by the fact that eugenol has been proven to have antiplatelet and antithrombotic effects, including platelet aggregation inhibition and thromboxane A_2_ synthesis inhibition (69). As Wang et al. (70) state, eugenol disrupts the activity of cyclooxygenase (COX-1) to suppress the production of arachidonic acid and its further neutrophilization. Moreover, its antioxidant effect suppresses reactive oxygen species (ROS) (71), which is commonly the main initiator of platelet activation in the scenario of diabetes and inflammatory stress. The lower levels of PLT in G_4_ and G_5_ are, therefore, probably some sort of a normalization of hyperactive platelet activity as opposed to an expression of disease pathology of thrombocytopenia.

Histopathological examination of treated skin specimens showed normalization of the epidermal and dermal structure, decreased cellular infiltration in the inflammatory cells, and organized collagen deposition, proving the healing potential of the CONE incorporated cream. These findings support the previous report by El-Zahaby et al. (11), while the CONE-based cream promoted tissue regeneration and curbed infections. The bioactives of clove act as antioxidants, reducing oxidative stress and supporting the restoration of normal skin structure (72). According to Banerjee et al. (64), a therapy via clove oil emulsion works effectively to achieve rapid wound healing, the skin layers, and production of collagen, which supports tissue repair. Similarly, Gul et al. (73) developed clove and olive oil NE to enhance skin penetration and tissue repair. Moreover, Mohamed et al. (65) also described histological improvement in systemic organs after treatment with CONE. Altogether, these results demonstrate the potential use of CONE to aid in skin regeneration through increased bioactive delivery to the wound site and reduction of oxidative damage.

### 4.8 Molecular docking

The molecular docking approach was used to explore the binding ability of eugenol to the major molecules participating in the human inflammatory process. *In-silico* studies indicated that eugenol tends to construct a stable complex with CRP and IL-1β, which underlies its anti-inflammatory activities. The docking of eugenol against CRP showed a favourable binding energy of −4.7 kcal/mol. CRP is a prototypical acute-phase reactant and a sensitive biomarker manifesting tight association with systemic inflammation; it also has direct pro-inflammatory actions such as complement activation and cytokine production (74). *π*–π T-shaped interaction with PHE66 and a π-alkyl interaction with LEU64 maintain the eugenol molecule in this important ligand-binding groove. The binding mode indicates that eugenol could be a competitive inhibitor that may obstruct the endogenous ligands binding and overlap with the inflammatory signaling of CRP, so as to play the role of inflammatory inhibitor (75). Notably, docking against 7PKB revealed π–π and π-alkyl interactions consistent with previously reported stabilization mechanisms for eugenol in inflammatory targets (e.g., COX-2, 5-LOX), where aromatic stacking with phenylalanine residues plays a dominant role in affinity enhancement (76).

Similarly, the simulation with Interleukin-1β (PDB ID: 9ILB), a primary cytokine driving fever and acute inflammation, showed a robust binding affinity (−4.9 kcal/mol). A conventional hydrogen bond with VAL41 provides directional stability, while hydrophobic contacts with LEU62 and VAL40 further secure the ligand in the protein’s hydrophobic cavity. The presence of a π-sulfur interaction with MET20 further enhances binding stability, analogous to eugenol’s interactions with PPARγ (77, 78), suggesting similar stabilization patterns. By occupying a key pocket on the IL-1β surface, eugenol could sterically hinder its interaction with the IL-1 receptor (IL-1R), thereby disrupting downstream pro-inflammatory signaling pathways such as NF-κB and MAPKs (79).

These molecular interactions provide a mechanistic rationale for eugenol’s documented ability to ameliorate inflammation by modulating multiple signaling proteins (2). The docking outcomes with 7PKB and 9ILB demonstrate that eugenol can engage critical inflammatory enzymes via both hydrophobic and hydrogen bonding interactions. This supports the potential use of eugenol, particularly in nanoemulsion formulations, to intercept and suppress inflammatory pathways *in-vivo*.

## 5 Conclusion

This study confirms that CONE-incorporated cream is a promising approach for the treatment of skin inflammation. The optimized NE with small droplet size, low Polydispersity Index and high entrapment efficiency displayed strong antifungal, antioxidant and anti-inflammatory activity. *In-vivo* application of CONE based cream in mice model normalized hematological parameters, inhibited inflammatory cell infiltration, and re-epithelialization of tissues as confirmed by histopathology. The sustained drug release and stability of the cream also demonstrate its clinical relevance. Taken together, these data demonstrated that the clove oil encapsulated in nanoemulsion improves bioavailability and efficacy with reduced risk for irritation, which could facilitate its further development as a safe and effective topical treatment. This study also has some limitations, which include a small sample size (*n* = 5), a lack of formal power assessment, quantitative toxicity or irritation scoring, and dose–response assessment *in vivo*. These limitations in future research can be overcome by diversifying the size of animal groups, conducting toxicological tests, and systematic dose–response studies to make a more comprehensive spectrum of the safety and effectiveness of the formulation.

## Data Availability

The raw data supporting the conclusions of this article will be made available by the authors, without undue reservation.

## Footnotes

1
https://www.rcsb.org/

2
https://pubchem.ncbi.nlm.nih.gov/

3
https://www.swissdock.ch/

## Glossary

CONE: clove oil nanoemulsion
CEO: clove essential oil
EO: essential oil
NE: nanoemulsion
PDI: Polydispersity Index
DLS: dynamic light scattering
TEM: transmission electron microscopy
GC–MS: gas chromatography–mass spectrometry
MIC: minimum inhibitory concentration
DPPH: 2,2-diphenyl-1-picrylhydrazyl
ABTS: 2,2′-azino-bis(3-ethylbenzothiazoline-6-sulfonic acid)
CMC: carboxymethyl cellulose
RBC: red blood cells
WBC: white blood cells
HGB: hemoglobin
HCT: hematocrit
MCV: mean corpuscular volume
MCH: mean corpuscular hemoglobin
MCHC: mean corpuscular hemoglobin concentration
PLT: platelets
MPV: mean platelet volume
PCT: plateletcrit
PDW: platelet distribution width
CRP: C-reactive protein
ANOVA: analysis of variance
RSM: response surface methodology
BBD: Box–Behnken Design
IL-1β: interleukin-1 beta
PDB: protein data bank
OD: optical density
UV–Vis: ultraviolet–visible spectroscopy
H&E: hematoxylin and eosin
